# Survey on the Use of Whole-Genome Sequencing for Infectious Diseases Surveillance: Rapid Expansion of European National Capacities, 2015–2016

**DOI:** 10.3389/fpubh.2017.00347

**Published:** 2017-12-18

**Authors:** Joana Revez, Laura Espinosa, Barbara Albiger, Katrin Claire Leitmeyer, Marc Jean Struelens, ECDC National Microbiology Focal Points and Experts Group, Ákos Tóth

**Affiliations:** ^1^European Centre for Disease Prevention and Control, Stockholm, Sweden

**Keywords:** whole-genome sequencing, genomic epidemiology, public health laboratory capacity, public health surveillance, outbreak investigation, food and waterborne infections, antimicrobial resistance, vaccine-preventable diseases

## Abstract

Whole-genome sequencing (WGS) has become an essential tool for public health surveillance and molecular epidemiology of infectious diseases and antimicrobial drug resistance. It provides precise geographical delineation of spread and enables incidence monitoring of pathogens at genotype level. Coupled with epidemiological and environmental investigations, it delivers ultimate resolution for tracing sources of epidemic infections. To ascertain the level of implementation of WGS-based typing for national public health surveillance and investigation of prioritized diseases in the European Union (EU)/European Economic Area (EEA), two surveys were conducted in 2015 and 2016. The surveys were designed to determine the national public health reference laboratories’ access to WGS and operational WGS-based typing capacity for national surveillance of selected foodborne pathogens, antimicrobial-resistant pathogens, and vaccine-preventable diseases identified as priorities for European genomic surveillance. Twenty-eight and twenty-nine out of the 30 EU/EEA countries participated in the survey in 2015 and 2016, respectively. National public health reference laboratories in 22 and 25 countries had access to WGS-based typing for public health applications in 2015 and 2016, respectively. Reported reasons for limited or no access were lack of funding, staff, and expertise. Illumina technology was the most frequently used followed by Ion Torrent technology. The access to bioinformatics expertise and competence for routine WGS data analysis was limited. By mid-2016, half of the EU/EEA countries were using WGS analysis either as first- or second-line typing method for surveillance of the pathogens and antibiotic resistance issues identified as EU priorities. The sampling frame as well as bioinformatics analysis varied by pathogen/resistance issue and country. Core genome multilocus allelic profiling, also called cgMLST, was the most frequently used annotation approach for typing bacterial genomes suggesting potential bioinformatics pipeline compatibility. Further capacity development for WGS-based typing is ongoing in many countries and upon consolidation and harmonization of methods should enable pan-EU data exchange for genomic surveillance in the medium-term subject to the development of suitable data management systems and appropriate agreements for data sharing.

## Introduction

In the European Union (EU), surveillance of 53 communicable diseases, healthcare-associated infections and antimicrobial resistance is conducted jointly by the European Centre for Disease Prevention and Control (ECDC) and the member states based on national case notification in accordance with EU case-definitions which are combining clinical and laboratory criteria (1). In addition, voluntary reporting to ECDC of molecular typing data on selected infectious agents and antimicrobial resistance determinants is encouraged for enhanced surveillance and epidemic response (2). Many EU countries use molecular typing methods, such as pulsed-field gel electrophoresis, multilocus variable number tandem repeat analysis (MLVA), and gene sequencing. Typing results are then shared in quality-assured, standard format on a voluntary basis for EU-level surveillance and control of diseases and drug-resistant pathogens, including foodborne infections and drug-resistant tuberculosis (2–4). However, it is patent that the effectiveness of interventions for the control of communicable diseases is limited by the lower resolution of these molecular typing methods compared to that of genomic analysis with next-generation sequencing (NGS) (5–10). Additional advantages of whole-genome sequencing (WGS)-based typing for supporting public health include its higher accuracy for tracing transmission and identifying infection sources, high reproducibility, timeliness, and throughput (5–7, 9, 10). As the technology progresses, it is becoming increasingly efficient and cost-competitive for diagnostic and surveillance purposes (9–12). For instance, WGS-derived resistome prediction for *Mycobacterium tuberculosis* was found to be 93% accurate to detect and characterize multidrug-resistant (MDR) tuberculosis cases with a median reporting of 21 days and at 7% lower cost than culture-based methods (9). Despite these advantages, current costs of implementation of NGS and lack of expertise as well as the need for adapting epidemiological investigation methods may limit its use by public health laboratories (8, 10). In addition, further harmonization for bioinformatic analysis, smart information technology solutions for WGS data storage and sharing, and trained staff with new skill mix are fundamental elements to translate genomic epidemiology into real-life infection control and prevention (5, 8, 13).

Several NGS platforms using diverse sequencing technologies are currently available. Even with limited knowledge of bioinformatics, it is possible to use these platforms for diagnostic purposes, using available user-friendly software packages, either commercial or open source (5, 13). Several public health laboratories have developed and validated in-house pipelines which will require harmonization to generate fully reproducible and comparable data between laboratories at local, (inter)regional, and international scales. In particular, the breadth and depth of sequence coverage, the data cleaning and analysis processes [including sequence assembly, alignment, filtering, mapping, and single-nucleotide polymorphism (SNP) and allele calling], the reference genomes and genomic similarity cut-off values and reference nomenclature for the typing schemes, must be agreed upon (5, 8, 9, 13). In addition, external quality assessments have to be further developed for verifying effective harmonization of WGS data analysis for public health (5, 13). Public data sharing is desirable as it contributes wider population baseline data for the detection of emerging infectious diseases and allows independent reanalysis of sequences to generate new knowledge (7, 9). Reaching this goal will require adopting appropriate data transfer agreements that protect legitimate intellectual property rights (14).

The state of the art evolves toward WGS as replacement of other molecular methods for surveillance and outbreak investigations (2, 5–9, 13). Taking stock of the latest advances (6), the ECDC has outlined a priority list of diseases for which to gradually integrate WGS data into EU-level surveillance systems and multi-country investigations of cross-border outbreaks (2, 6). This ambitious European cooperative process builds upon the operational capacity to implement WGS- based typing for public health applications among Member States of the EU and European Economic Area (EEA) (2). To assess the EU/EEA Member States national capacities to implement WGS-based typing, ECDC performed a web-based questionnaire survey in two consecutive years, 2015 and 2016, mapping (i) access of national public health reference laboratories (NRL) to NGS technologies and bioinformatics expertise and (ii) use by these laboratories of WGS-based typing for national surveillance and outbreak investigations. Diseases covered in the survey were the eight uppermost priority foodborne, antimicrobial–resistant, and vaccine-preventable pathogens selected for European genomic surveillance.

## Materials and Methods

European Centre for Disease Prevention and Control used the online survey software (https://ec.europa.eu/eusurvey/) for the collection of relevant information by the National Microbiology Focal Points (NMFP) nominated by public health authorities from the 30 EU/EEA countries. The survey collected information on WGS practice and development plans as of July 2015 and July 2016 by the competent NRL. Invitation to answer the first survey was sent on 29 July 2015 and it was open until 13 October 2015, and the second survey invitation was sent on 28 July 2016 with a deadline set for 11 November 2016 (Data S1 in Supplementary Material). The survey contained 46 questions covering public health NRL access to WGS, bioinformatics expertise and WGS-based operational typing capacity and practice for outbreak investigations and/or national surveillance for eight pathogens prioritized for European genomic surveillance, including foodborne pathogens [*Listeria monocytogenes, Salmonella enterica*, and Shiga toxin-producing *Escherichia coli* (STEC)], antimicrobial-resistant pathogens (carbapenemase-producing *Enterobacteriaceae* (CPE), antibiotic-resistant *Neisseria gonorrhoeae* and MDR *M. tuberculosis*), and vaccine-preventable diseases (*Neisseria meningitidis* and human influenza virus). In the 2016 survey, additional questions included whether WGS was used as first-line typing method or as second-line typing method complementary to results obtained with other molecular typing methods, as well as describing the sampling frame used for WGS-based typing, bioinformatic analysis and data storage methods. For both surveys, two reminders were sent for the data collection and two validation phases. All authors approved the present manuscript and data presentation by country.

## Results

### National Public Health Reference Laboratory Access to NGS Technologies for Public Health Operations

Twenty-eight and 29 of the 30 EU/EEA countries replied to the survey in 2015 and 2016, respectively. In 2015, one or more NRL reported access to NGS technology for public health operations in 22/28 countries. One year later, NRL in 25/29 countries had access to NGS, either as internal (*n* = 12), external (*n* = 6) or both services (*n* = 7). Eighteen and 22 countries reported which technologies were used in 2015 and 2016, respectively; the most frequently used was Illumina followed by Ion Torrent technology (Table 1). Four and three countries were unable to list the technologies in 2015 and 2016, respectively, as the access was limited to external services only and on an *ad hoc* basis. In both years, three countries that did not have access to NGS were planning to implement WGS-based typing by 2018 for pathogens listed as EU priorities. Lack of funding, staff, and expertise was the reasons stated by NRL in countries with limited or no access to NGS technology.

**Table 1:** Number of EU/EEA countries with one or more national public health reference laboratories having access to next-generation sequencing (NGS) technologies for routine public health operations, by technology and instrument used, 2015–2016.

NGS technology	Instrument	Number of countries with access to NGS	
		2015 (*n* = 22)	2016 (*n* = 25)
Illumina	MiniSeq	–	2
	MiSeq series	13	17
	NextSeq	2	5
	HiSeq series	6	8
Ion Torrent	PGM	5	6
	Proton	2	1
	S5XL	–	1
Pacific Biosciences—PacBio	PacBio RSII	2	1
Oxford Nanopore Technologies	MinION	–	2
Applied Biosystems Inc—SOLiD	ABI SOLiD	1	–
Not specified/reported	–	4	3

### WGS-Based Typing Capabilities for Surveillance and Outbreak Investigations

**Figure 1:**
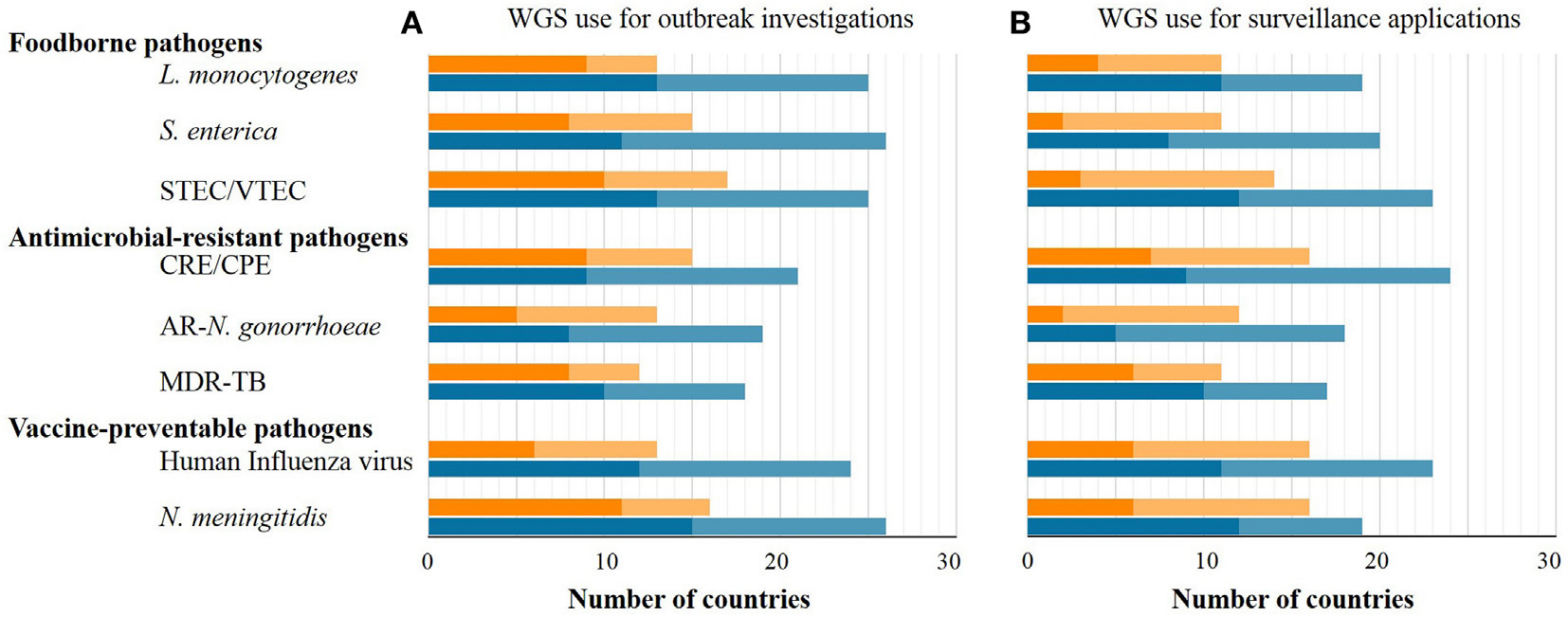
Number of EU/EEA countries with capability to use whole-genome sequencing (WGS)-based typing (dark tone) or planning to use it by 2018 (light tone) as of mid-2015 (orange bars) and mid-2016 (blue bars) applied to outbreak investigations **(A)** or surveillance **(B)**, by disease group and pathogen.

**Figure 2:**
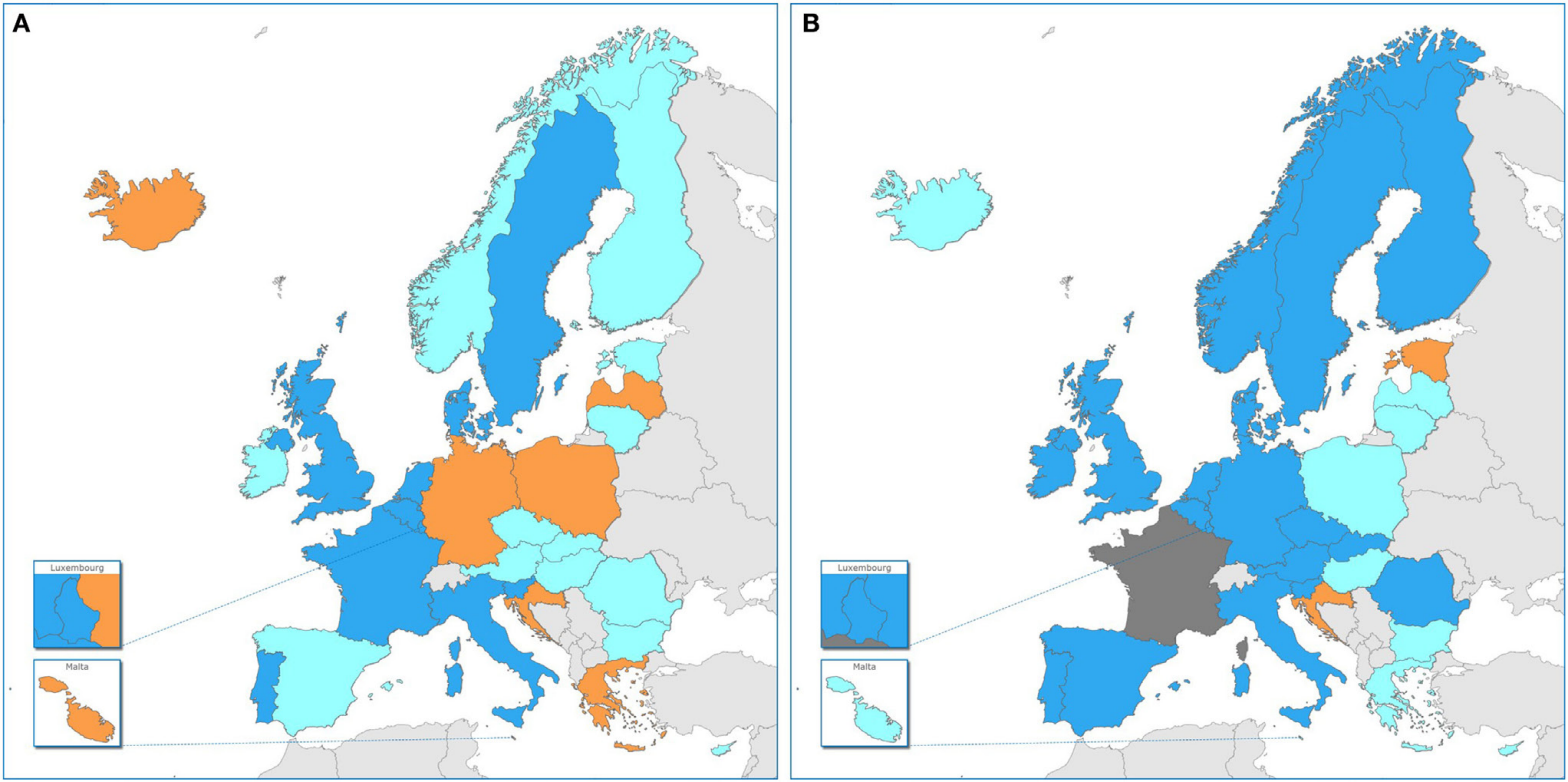
Use of whole-genome sequencing (WGS)-based typing of human pathogens in Public Health Reference Laboratories for routine national surveillance by country in the EU/EEA, 2015–16. Dark blue: WGS-based typing used routinely of at least one human pathogen; light blue: national plan in place/in progress for WGS-based typing for surveillance of at least one human pathogen by 2018; orange: no use in 2016 nor national plan for use by 2018; dark gray: no information. **(A)** 2015 data, cross-validated with EULabCap report (15) and **(B)** 2016 data.

The number of EU/EEA countries reporting NRL capability with WGS-based typing increased markedly over 1 year (July 2015–July 2016) as applied to both outbreak investigation and surveillance (Figure 1). WGS-based typing was used to support outbreak investigations for at least one pathogen in 18 countries in 2015 and in 23 countries in 2016, a relative 1.3-fold increase within 1 year (Figure 1). Use of WGS-based typing to support communicable disease surveillance for at least one pathogen also increased over this one-year period from 10 to 16 countries, respectively (Figures 1 and 2), a relative 1.6-fold increase. The magnitude of annual expansion in the number of countries using WGS for surveillance varied between 1.2- and 4.0-fold increase depending on the disease under surveillance. In addition, more non-user countries reported that they had started planning to implement WGS-based typing by 2018 for these applications between survey years (Figures 1 and 2).

The target pathogens for which countries most frequently used WGS-based typing in 2015 and 2016 for both outbreak and surveillance applications were, in order of decreasing frequency, *N. meningitidis* followed by STEC and *L. monocytogenes* (Figure 1). In 2016, 15 EU/EEA countries used WGS-based typing for national surveillance of human infections with antimicrobial-resistant pathogens in the survey, with 10, 9, and 5 countries using it for surveillance of MDR-*M. tuberculosis*, (CPE), and antibiotic-resistant *N. gonorrhoeae*, respectively (Figure 1).

Regarding national development plans for outbreak investigations, the pathogens which the largest number of countries were planning to characterize by WGS by 2018 are *N. meningitidis* followed by *S. enterica, L. monocytogenes*, and STEC. CPE were predicted to become the most frequent surveillance target by 2018 for WGS-based typing across the EU/EEA, followed by STEC, *S. enterica, L. monocytogenes*, and *N. meningitidis* (Figure 1).

### WGS Typing Scheme, Sampling Frame, Data Analysis, and Storage Used by NRLs in 2016

The WGS-based typing scheme, sampling frame, bioinformatic analysis, and data storage practice used by NRLs were surveyed in 2016 (Table 2). WGS was used as first-line, standalone typing method most frequently for the characterization of STEC followed by *L. monocytogenes* and *N. meningitidis* (Table 2). These pathogens were also the most frequent ones that were WGS-typed following a comprehensive sampling. For *S. enterica* and influenza virus, which a substantial number of countries typed by WGS for national surveillance, it was predominantly used as second-line typing method and/or limited to a subset of available samples (Table 2).

**Table 2:** Number of EU/EEA countries using WGS-based typing in the National Public Health Reference Laboratories and respective typing scheme, sampling frame, bioinformatic analysis, and raw data storage by disease group and pathogen, 2016.

	Foodborne pathogens			Antimicrobial-resistant pathogens			Vaccine-preventable pathogens	

	*L. monocytogenes*	*S. enterica*	Shiga toxin-producing ***E. coli*** (STEC)	carbapenemase-producing *Enterobacteriaceae* (CPE)	AR *N. gonorrhoeae*	multidrug-resistant (MDR) *M. tuberculosis*	Human Influenza virus	*N. meningitidis*

Number of countries using WGS-based typing	13	11	13	9	5	10	12	15
**Typing scheme**								
First-line	6	3	7	5	5	5	2	6
Second-line	7	8	6	4	–	5	10	9
**Sampling frame**								
Continuous comprehensive	8	1	6	2	–	8	–	9
Sentinel/subset of samples	4	10	7	7	5	2	12	6
**Bioinformatic analysis^a^**								
Core genome multi-locus sequence typing	10	8	5	5	4	4	–	11
Single-nucleotide polymorphism	7	8	8	4	3	8	4	4
Resistome prediction	3	6	7	8	–	9	10	4
wgMLST	2	2	4	3	3	1	–	–
Virulome/mobilome prediction	1	4	11	4	1	–	–	–
MLST prediction	3	1	2	–	–	–	–	–
Serogroup prediction	3	2	3	–	–	–	–	–
NG-MAST	–	–	–	–	4	–	–	–
Speciation	–	–	–	–	–	1	–	–
Hemagglutinin and neuraminidase sequence prediction	–	–	–	–	–	–	9	–
Phylogenetic relationship	–	–	–	–	–	–	10	–
Identification of specific point mutations	–	–	–	–	–	–	10	–
rMLST	–	–	–	–	–	–	–	5
MLST + *porA* VR1 and VR2 + *fetA*	–	–	–	–	–	–	–	13
Vaccine antigens prediction	–	–	–	–	–	–	–	3
Other not specified	–	–	–	–	–	–	–	2
**Raw sequence data storage**								
Dedicated closed database(s)	11	9	11	8	4	9	9	10
Publicly available database(s)	1	1	–	–	–	–	1	3
Both dedicated closed database(s) and publicly available database(s)	1	1	2	1	1	1	2	2

*^a^Not mutually exclusive*.

Among the antimicrobial-resistant pathogens surveyed, MDR *M. tuberculosis* was the most intensively WGS-based typed by NRL in most countries using the technology as first-line typing method on a continuous comprehensive sample of reported cases (Table 2). By contrast, the majority of countries using WGS-based as first-line method typing for surveillance of CPE or carbapenem-resistant *Enterobacteriacea* (CRE) or antibiotic-resistant *N. gonorrhoeae*, restricted typing to a sentinel subset of samples (Table 2).

The bioinformatics expertise and competence available in house to NRL for routine WGS data analysis in 2016 were reported as sufficient in only three countries whereas in 16 countries NRL using WGS had only a partial degree of competence supplemented with external expertise; and in the remaining countries, analysis was fully outsourced to external services. Among diverse bioinformatic pipelines used by NRL for WGS data analysis, the core genome multi-locus sequence typing (cgMLST), often used in combination with SNPs analysis, was the most commonly used approach across pathogens (Table 2). As expected, bioinformatic analyses were intrinsically dependent on the pathogen typed: while for the foodborne pathogens *L. monocytogenes* and *S. enterica*, cgMLST and SNP analysis were the most frequently used, virulome/mobilome prediction was used the most for STEC typing. WGS-based resistome prediction was commonly used for typing CPE/CRE, MDR-*M. tuberculosis* and human influenza virus. Finally, the bioinformatic analysis and typing schemes most commonly used for characterizing *N. meningitidis* were MLST + *porA* VR1 and VR2 + *fetA* as well as cgMLST allelic nomenclature (Table 2).

For WGS data storage, the vast majority of EU/EEA countries deposited the raw sequence (fastq) data produced by the NRL on dedicated closed databases (either national or international). The most frequently reported reason for this practice was the priority given to use this information for national reporting and risk assessment, followed by priority to permit scientific publication of original data and lastly for personal data protection. In 2016 raw sequence data were seldom deposited in publicly available databases (e.g., European Nucleotide Archive) with only three to five countries doing so for human influenza virus and *N. meningitidis*, respectively, and only one or two countries releasing data for any other pathogens under survey (Table 2).

## Discussion

The rapid transformation from molecular to genomic epidemiology of infectious diseases is opening a new era of “precision public health” by unveiling the detailed transmission dynamics of infection and antimicrobial resistance and thereby enabling more effective and better targeted control interventions (5, 6, 8, 9, 12, 13). Fulfilling its mandate to collate, appraise, and disseminate information for public health action, ECDC is committed to foster the integration of WGS-based typing for infectious disease surveillance and outbreak investigations at European level (6, 12). This implies harmonizing surveillance methods and keeping pace with the different stages of WGS-based typing implementation among European public health reference laboratories (2). To this end, we have undertaken to monitor the transition to NGS technologies through annual surveys with our public health partners across the EU/EEA. To our knowledge, this is the first assessment of the national capacities and use of WGS-based typing in public health microbiology in Europe.

It is noteworthy that by 2016, the NRL in 25 EU/EEA countries, or rather 26 countries taking into account one country reporting capability in 2015 but not participating in the 2016 survey, had access to WGS-based typing for their routine public health applications. Illumina technology was the most frequently used platform, followed by Ion Torrent technology. This technology distribution is in accordance with that found by a recent survey conducted among research, food safety and public health institutions worldwide (7). More importantly, we found that by 2016 more than half of EU and EEA countries had moved to routine use of WGS-based typing data for national surveillance, whereas none had such operational capability in 2013 and the number of countries implementing it has increased twofold between 2014 and 2016 (15). This rapid pace of innovation in public health laboratories across countries supports the ECDC vision of pan-European surveillance systems sharing WGS-based typing data for key diseases by 2020 (6). The present study indicated disparity of practice among reference laboratories in Europe (Figure 2), with some performing NGS on a limited basis, e.g., for outbreak investigations, while others are applying WGS-based typing on a much larger scale, e.g., for near real-time surveillance and outbreak detection, as previously reported at national level (3, 6, 13, 16–20). This diversity of practice among countries may be partly linked to restriction to service capacity related to test costs (7, 21) or, as identified in the herein study, lack of trained staff with sufficient bioinformatics expertise. Additional country determinants of WGS capacity for public health services may include variation in the national health expenditure per capita, public health microbiology system capacity and investment in translational health research and innovation (12, 15).

The sampling and typing modalities for a given national genomic surveillance program depend upon the surveillance objectives specific for a particular disease and its local epidemiology and public health importance (2). As compared to the previous gold-standard typing methods used with food-borne disease surveillance, early and more sensitive outbreak detection can be achieved through first-line WGS-based genotyping to identify clusters of genetically related isolates, as recently shown by nationwide proof of concept studies (10, 12, 22). The results presented here show that this demanding approach of comprehensive sampling for WGS-based typing was still the exception rather than the rule in 2016 among the 16 EU/EEA countries where it was used as part of national surveillance programs. Structured sentinel surveys offer an alternative approach which is especially suitable for the surveillance of MDR *Enterobacteriaceae* and *N. gonorrhoeae* at European scale, combining the analysis of strain genomic type and antimicrobial resistance phenotype with epidemiological risk factors to monitor the emergence and delineate the routes of spread of MDR clones and genetic determinants (2, 12, 23–25). In the present study, this sentinel approach was also shown to be the preferred sampling frame used for genomic surveillance of antimicrobial resistance at EU member state level.

It is encouraging to note that the diseases and drug resistance issues targeted for WGS-based typing by national surveillance programs, as described here, match well the mid-term priorities for EU genomic surveillance (2). Despite common public health priorities and surveillance targets, different NGS instruments and multiple bioinformatic analysis pipelines were being used across the EU/EEA laboratories, a mixed practice which is not surprising since these platforms and tools are still undergoing continuous improvements and field trial testing. Nevertheless, it is noticeable that cgMLST nomenclatures were broadly used among these laboratories to assign genomic types to bacterial pathogens (26) in accordance with recent guidelines on genomic surveillance standards for foodborne diseases (6, 21, 27). Therefore, WGS-based genotype data portability between different NGS platforms and analytical pipelines appears feasible in the short term. There are different computational approaches to predict antibiotic resistance from WGS data, the simplest by mapping of the sequence reads against a reference database of resistance genes or mutations, scoring the absence or presence of these factors, and predicting a resistance profile accordingly (9). However, the establishment of curated knowledge bases on drug resistance genetic determinants will be necessary to overcome the quality gaps in published pheno-genotype correlations that are currently hampering the accuracy of susceptibility phenotype predictions from WGS data (28). For tuberculosis, progress to bridge this gap is well advanced making WGS-based diagnostics and drug resistance detection a potential tool to improve clinical management and control of the disease in the near future (11, 13).

The rapid expansion of WGS technology in public health laboratories is paralleled with its gradual introduction in clinical microbiology laboratories, where technology evaluation studies show great promise to identify and characterize pathogens and detect, investigate, and control transmission of multi-drug epidemic strains in healthcare settings with increased timeliness and accuracy (8, 13). In the future, decentralized molecular diagnostic testing and WGS analysis will challenge the traditional model of clinical sample referral to public health laboratories for specialist typing as part of surveillance activities. Technical standardization and collaboration between the clinical and public health actors will be key to ensure quality and portability of WGS-derived data across integrated laboratory information systems for surveillance purposes.

We noted that, for practical reasons, the majority of the NRL in EU/EEA countries deposited raw WGS data in closed databases. This can be counterproductive, as such publicly shared data linked to minimal epidemiological metadata can generate new knowledge and may facilitate prevention of infectious diseases (7). To fully utilize the potential of WGS, open access pan-EU or global databases need to be implemented for sharing the WGS data and minimum clinical, epidemiological, and other contextual metadata. Therefore, practical solutions must be sought that enable open access to valuable biological information for further biological and public health research while safeguarding legitimate data protection and ownership.

Further development, critical evaluation and harmonized application of WGS-based typing solutions for public health protection can only be delivered through engaging intersectorial and international collaborations. These joint efforts currently involve the close collaboration between ECDC and the European Food Safety Authority toward One-health interoperable systems for the molecular surveillance of zoonotic pathogens and drug resistance, as well as partnership with relevant EU research projects and global initiatives (e.g., PulseNet International, Global Microbial Identifier) (6).

A study limitation relates to the semantic ambiguity of terms used for the questionnaire, such as the distinction between “control-oriented surveillance” versus “policy-oriented surveillance,” or the distinction between “outbreak detection,” as a possible output of surveillance, and “outbreak investigation” as a follow-up action. The risk of such ambiguities was mitigated by providing a glossary with definitions of terms with the questionnaire and helpdesk support to participants to clarify questions by bilateral discussion if needed. A second limitation of accuracy of the data is related to the complexity and fluidity of national technical capacities collected by each national data collector, using a 6-month arbitrary time period as snap-shot window on a continuing development process.

In conclusion, our study established that the vast majority of NRL in EU/EEA countries had access to microbial pathogen WGS-based typing by mid-2016 and used it widely for public health investigations of infection and drug resistance transmission. Over a short 2-year time span after its introduction, a rapid shift toward implementation of the technology was manifest across the EU/EEA with half the countries routinely using WGS for national surveillance in 2016. Further WGS use is planned in many countries and should enable pan-EU data exchange in the medium term, subject to pipeline compatibility and agreed nomenclature and data management. The findings of this survey suggest that key capacity gaps include expertise in epidemiological-WGS data integrative analysis and user-friendly international nomenclature. Together with its EU and international partners ECDC will contribute to broaden capacities in these areas along national public health priorities with the primary aim to facilitate inter-operability with EU surveillance and outbreak response programs.

## ECDC National Microbiology Focal Points and Experts Group

The following Authors, who are listed in alphabetical order, contributed to the work of the ECDC National Microbiology Focal Points and Experts Group: **Ákos Tóth**, National Center for Epidemiology, Budapest, Hungary; **Algirdas Griškevičius**, National Public Health Surveillance Laboratory, Vilnius, Lithuania; **Alkiviadis Vatopoulos**, National School of Public Health, Athens, Greece; **Anna Skoczynska**, National Medicines Institute, Warsaw, Poland; **Annalisa Pantosti**, Istituto Superiore di Sanità, Rome, Italy; **Bruno Coignard**, Santé publique France, Saint-Maurice, France; **Cyril Klement**, Regional Public Health Authority, Banská Bystrica, Slovakia; **Despo Pieridou**, Nicosia General Hospital, Nicosia, Cyprus; **Dominique Caugant**, Norwegian Institute of Public Health, Oslo, Norway; **Eleanor McNamara**, Health Service Executive, Public Health Laboratory, Dublin, Ireland; **Franz Allerberger**, Austrian Agency for Health and Food Safety, Vienna, Austria; **Gabriel Ionescu**, National Institute of Research and Development for Microbiology and Immunology “Cantacuzino”, Bucharest, Romania; **Graziella Zahra**, Pathology Laboratory, Msida, Malta; **Guido Werner**, Robert Koch Institut, Wernigerode, Germany; **Iva Christova**, National Centre of Infectious and Parasitic Diseases, Sofia, Bulgaria; **Joël Mossong**, National Health Laboratory, Luxembourg, Luxembourg; **Jonathan Green**, Public Health England, Liverpool, United Kingdom; **Jorge Machado**, Instituto Nacional de Saúde Doutor Ricardo Jorge, Lisbon, Portugal; **Julio Vazquez Moreno**, Centro Nacional de Microbiologia, Madrid, Spain; **Karl Kristinsson**, Landspitali University Hospital, Reykjavik, Iceland; **Mattias Mild**, Folkhälsomyndigheten, Solna, Sweden; **Metka Paragi**, Centre for Medical Microbiology, Ljubljana, Slovenia; **Nico Meessen**, National Institute for Public Health and the Environment, Bilthoven, Netherlands; **Oksana Savicka**, Riga East University hospital, Riga, Latvia; **Pavla Křížová**, National Institute of Public Health, Prague, Czechia; **Rita Peetso**, Health Board, Central, Tallinn, Estonia; **Saara Salmenlinna**, National Institute for Health and Welfare, Helsinki, Finland; **Steven Van Gucht**, Scientific Institute of Public Health, Brussels, Belgium; **Thea K Fischer**, Statens Serum Institut, Copenhagen, Denmark; **Vera Katalinić-Janković**, Croatian Institute of Public Health, Zagreb, Croatia.

## Author Contributions

JR: questionnaire design, data collection, validation and analysis, and writing the first draft. LE: questionnaire design and piloting, data collection, validation and analysis, and draft review. BA and KL: review of questionnaire and first draft. MS: study and questionnaire design, analysis plan, and rewriting the final draft. ECDC NMFP group: data collection, validation, and draft review.

## Conflict of Interest Statement

The authors declare that the research was conducted in the absence of any commercial or financial relationships that could be construed as a potential conflict of interest.

## References

[1] European Commission. Decision No 2012/506/EU of the Commission of 8 August 2012 Amending Decision 2002/253/EC Laying Down Case Definitions for Reporting Communicable Diseases to the Community Network Under Decision No 2119/98/EC of the European Parliament and of the Council (Notified Under Document C(2012) 5538). (2012). Available from: http://eur-lex.europa.eu/LexUriServ/LexUriServ.do?uri=OJ:L:2012:262:0001:0057:EN:PDF

[2] European Centre for Disease Prevention and Control. ECDC Roadmap for Integration of Molecular and Genomic Typing into European-Level Surveillance and Epidemic Preparedness. Version 2.1, 2016–2019. Stockholm: ECDC (2016). Available from: http://ecdc.europa.eu/en/publications/Publications/molecular-typing-EU-surveillance-epidemic-preparedness-2016-19-roadmap.pdf

[3] Fonteneau L Jourdan Da Silva N Fabre L Ashton P Torpdahl M Muller L Multinational outbreak of travel-related *Salmonella* Chester infections in Europe, summers 2014 and 2015. Euro Surveill (2017) 22(7):30463. 10.2807/1560-7917.ES.2017.22.7.30463 28230522 PMC5322187

[4] Nikolayevskyy V Hillemann D Richter E Ahmed N van der Werf MJ Kodmon C External quality assessment for tuberculosis diagnosis and drug resistance in the European Union: a five year multicentre implementation study. PLoS One (2016) 11(4):e0152926. 10.1371/journal.pone.0152926 27055064 PMC4824391

[5] Sintchenko V Holmes EC. The role of pathogen genomics in assessing disease transmission. BMJ (2015) 350:h1314. 10.1136/bmj.h1314 25964672

[6] European Centre for Disease Prevention and Control. Expert Opinion on Whole Genome Sequencing for Public Health Surveillance. Stockholm: ECDC (2016). Available from: http://ecdc.europa.eu/en/publications/_layouts/forms/Publication_DispForm.aspx?List=4f55ad51-4aed-4d32-b960-af70113dbb90&ID=1555

[7] Moran-Gilad J Sintchenko V Pedersen SK Wolfgang WJ Pettengill J Strain E Proficiency testing for bacterial whole genome sequencing: an end-user survey of current capabilities, requirements and priorities. BMC Infect Dis (2015) 15:174. 10.1186/s12879-015-0902-3 25887164 PMC4392855

[8] Tang P Croxen MA Hasan MR Hsiao WW Hoang LM. Infection control in the new age of genomic epidemiology. Am J Infect Control (2017) 45(2):170–9. 10.1016/j.ajic.2016.05.015 28159067

[9] Schurch AC van Schaik W. Challenges and opportunities for whole-genome sequencing-based surveillance of antibiotic resistance. Ann N Y Acad Sci (2017) 1388(1):108–20. 10.1111/nyas.13310 28134443

[10] Moura A Tourdjman M Leclercq A Hamelin E Laurent E Fredriksen N Real-time whole-genome sequencing for surveillance of *Listeria monocytogenes*, France. Emerg Infect Dis (2017) 23(9):1462–70. 10.3201/eid2309.170336 28643628 PMC5572858

[11] van Soolingen D Jajou R Mulder A de Neeling H. Whole genome sequencing as the ultimate tool to diagnose tuberculosis. Int J Mycobacteriol (2016) 5(Suppl 1):S60–1. 10.1016/j.ijmyco.2016.10.036 28043616

[12] Struelens MJ Brisse S From molecular to genomic epidemiology: transforming surveillance and control of infectious diseases. Euro Surveill (2013) 18(4):20386 10.2807/ese.18.04.20386-en 23369387

[13] Deurenberg RH Bathoorn E Chlebowicz MA Couto N Ferdous M Garcia-Cobos S Application of next generation sequencing in clinical microbiology and infection prevention. J Biotechnol (2017) 243:16–24. 10.1016/j.jbiotec.2016.12.022 28042011

[14] Aarestrup FM Koopmans MG. Sharing data for global infectious disease surveillance and outbreak detection. Trends Microbiol (2016) 24(4):241–5. 10.1016/j.tim.2016.01.009 26875619

[15] European Centre for Disease Prevention and Control. EU Laboratory Capacity Monitoring System (EULabCap) – Report on 2015 Survey of EU/EEA Capabilities and Capacities. Stockholm: ECDC (2017). Available from: https://ecdc.europa.eu/sites/portal/files/documents/EULabCap_report-for-2015.pdf

[16] Inns T Ashton PM Herrera-Leon S Lighthill J Foulkes S Jombart T Prospective use of whole genome sequencing (WGS) detected a multi-country outbreak of *Salmonella* Enteritidis. Epidemiol Infect (2017) 145(2):289–98. 10.1017/S0950268816001941 27780484 PMC9507544

[17] Ashton PM Nair S Peters TM Bale JA Powell DG Painset A Identification of *Salmonella* for public health surveillance using whole genome sequencing. PeerJ (2016) 4:e1752. 10.7717/peerj.1752 27069781 PMC4824889

[18] Oteo J Perez-Vazquez M Bautista V Ortega A Zamarron P Saez D The spread of KPC-producing *Enterobacteriaceae* in Spain: WGS analysis of the emerging high-risk clones of *Klebsiella pneumoniae* ST11/KPC-2, ST101/KPC-2 and ST512/KPC-3. J Antimicrob Chemother (2016) 71(12):3392–9. 10.1093/jac/dkw321 27530752 PMC5890657

[19] Gillesberg Lassen S Ethelberg S Bjorkman JT Jensen T Sorensen G Kvistholm Jensen A Two *Listeria* outbreaks caused by smoked fish consumption-using whole-genome sequencing for outbreak investigations. Clin Microbiol Infect (2016) 22(7):620–4. 10.1016/j.cmi.2016.04.017 27145209

[20] Bukovski S Vacca P Anselmo A Knezovic I Fazio C Neri A Molecular characterization of a collection of *Neisseria meningitidis* isolates from Croatia, June 2009 to January 2014. J Med Microbiol (2016) 65(9):1013–9. 10.1099/jmm.0.000320 27452726

[21] European Centre for Disease Prevention and Control. Expert Opinion on the Introduction of Next-Generation Typing Methods for Food- and Waterborne Diseases in the EU and EEA. Stockholm: ECDC (2015). Available from: http://ecdc.europa.eu/en/publications/Publications/food-and-waterborne-diseases-next-generation-typing-methods.pdf

[22] Kwong JC Mercoulia K Tomita T Easton M Li HY Bulach DM Prospective whole-genome sequencing enhances national surveillance of *Listeria monocytogenes*. J Clin Microbiol (2016) 54(2):333–42. 10.1128/JCM.02344-15 26607978 PMC4733179

[23] Chisholm SA Unemo M Quaye N Johansson E Cole MJ Ison CA Molecular epidemiological typing within the European Gonococcal Antimicrobial Resistance Surveillance Programme reveals predominance of a multidrug-resistant clone. Euro Surveill (2013) 18(3):20358 10.2807/ese.18.03.20358-en 23351652

[24] Khong WX Xia E Marimuthu K Xu W Teo YY Tan EL Local transmission and global dissemination of New Delhi Metallo-Beta-Lactamase (NDM): a whole genome analysis. BMC Genomics (2016) 17:452. 10.1186/s12864-016-2740-0 27297071 PMC4906610

[25] European Centre for Disease Prevention and Control. ECDC Study Protocol for Genomic-Based Surveillance of Carbapenem-Resistant and/or Colistin-Resistant Enterobacteriaceae at the EU Level. Stockholm: ECDC (2017). Available from: http://ecdc.europa.eu/en/publications/Publications/Protocol-genomic-surveillance-resistant-Enterobacteriaceae.pdf

[26] Jolley KA Maiden MC. Automated extraction of typing information for bacterial pathogens from whole genome sequence data: *Neisseria meningitidis* as an exemplar. Euro Surveill (2013) 18(4):20379. 10.2807/ese.18.04.20379-en 23369391 PMC3977036

[27] Nadon C Van Walle I Gerner-Smidt P Campos J Chinen I Concepcion-Acevedo J PulseNet international: vision for the implementation of whole genome sequencing (WGS) for global food-borne disease surveillance. Euro Surveill (2017) 22(23):30544 10.2807/1560-7917.ES.2017.22.23.30544 28662764 PMC5479977

[28] Ellington MJ Ekelund O Aarestrup FM Canton R Doumith M Giske C The role of whole genome sequencing in antimicrobial susceptibility testing of bacteria: report from the EUCAST Subcommittee. Clin Microbiol Infect (2017) 23(1):2–22. 10.1016/j.cmi.2016.11.012 27890457

