# Supplementary material for: Survey on the Use of Whole-Genome Sequencing for Infectious Diseases Surveillance: Rapid Expansion of European National Capacities, 2015–2016
Source: Front Public Health. 2017 Dec 18;5:347. doi: 10.3389/fpubh.2017.00347 (PMC5741818; doi:10.3389/fpubh.2017.00347)
Supplement: Supplementary file 1 [file data_sheet_1.docx]

Supplementary Material

Survey on the use of Whole Genome Sequencing for infectious diseases surveillance: rapid expansion of European national capacities, 2015-2016

Joana Revez, Laura Espinosa, Barbara Albiger, Katrin Claire Leitmeyer, Franz Allerberger, Steven Van Gucht, Iva Christova, Vera Katalinić-Janković, Despo Pieridou, Pavla Křížová, Thea Fischer Kølsen, Rita Peetso, Saara Salmenlinna, Bruno Coignard, Guido Werner, Alkiviadis Vatopoulos, Ákos Tóth, Karl Kristinsson, Eleanor McNamara, Annalisa Pantosti, Oksana Savicka, Algirdas Griškevičius, Joël Mossong, Graziella Zahra, Nico Meessen, Dominique Caugant, Anna Skoczynska, Jorge Machado, Gabriel Ionescu, Cyril Klement, Metka Paragi, Julio Moreno Vazquez, Mattias Mild, Jonathan Green, Marc Jean Struelens*

*** Correspondence:** Marc Struelens, [Marc.Struelens@ecdc.europa.eu](mailto:Marc.Struelens@ecdc.europa.eu)

# Supplementary Data 1

*The questionnaire is available at* [*https://ec.europa.eu/eusurvey/runner/NGS_Survey_2016*](https://ec.europa.eu/eusurvey/runner/NGS_Survey_2016)


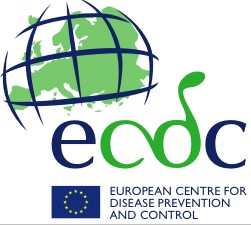
**NMFP questionnaire on national capacity for Whole Genome Sequencing (WGS) use for Public Health applications in EU/EEA countries.**

Fields marked with * are mandatory.

#
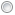

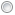

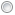

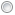

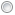

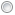

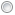

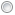

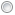

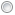

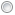

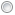

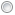

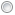

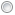

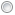
Country information

- Please specify the country you represent

| Austria | Belgium | Bulgaria | Croatia |
| --- | --- | --- | --- |
| Cyprus | Czech Republic | Denmark | Estonia |
| Finland | France | Germany | Greece |
| Hungary | Iceland | Ireland | Italy |
| Latvia | Lithuania | Luxembourg | Malta |
| Netherlands | Norway | Poland | Portugal |
| Romania | Slovakia | Slovenia | Spain |
| Sweden | United Kingdom |  |  |

-
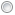

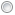

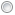

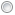

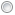

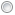

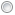

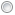

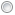

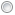

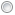

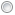

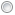

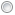
Please enter your name
- Please enter your email

# Current national NGS/WGS practices, as of July 2016

### This section is composed by three subsections (with a total of 6 questions): General questions (2 questions), current use of NGS technology for Public Health operations - applicable only if you have access to NGS (3 questions) and Mapping the Member States' training needs (1 question).

General Questions

- **1.** Do Public Health reference laboratories in your country have access to Next Generation Sequencing NGS technology for public health operations? (tick more than one if applicable; **if you only select the option "No", then please select Not applicable from questions 3-5)**


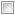
 Yes, internal access to NGS


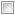
 Yes, external access to NGS (isolates or genetic material is sent elsewhere)
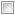
 No


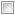
 Do not know

If external access to NGS, please specify where [e.g. other national laboratories, commercial provider(s)].

- **2.** If limited or no access, what is(are) the reason(s)? (tick more than one if applicable)


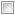
 Current plan to implement NGS/WGS within the next 3 years (2016-2018)
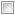
 No plan to implement NGS/WGS within the next 3 years (2016-2018)


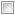
 Delayed implementation until further development of the application


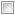
 Gaps/lack of expertise


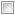
 Lack of funding


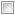
 Lack of staff to implement


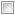
 Not applicable


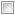
 Other

If other**,** please provide further details.

Current use of NGS technology for Public Health operations, as of July 2016

- **3.** Which NGS/WGS technology platform(s) is(are) used? (tick more than one, if applicable)


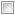
 Illumina MiniSeq
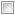
 Illumina MiSeq series


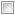
 Illumina NextSeq
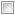
 Illumina HiSeq series


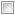
 Illumina HiSeq X series
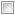
 Pacific Bioscience PacBio RS II
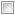
 Ion Torrent PGM
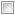
 Ion Torrent Proton


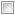
 Oxford Nanopore MinION
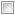
 Other
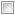
 Not applicable

If other**,** please specify which NGS/WGS technology platform(s) is used.

**4.** Do public health reference laboratories have access to sufficient bioinformatics expertise and

competence for routine WGS data analysis?


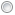
 Yes, sufficient bioinformatics expertise and competence within public health reference laboratories
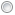
 Yes, some degree of expertise and competence supplemented with external expertise


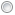
 Yes, fully outsourced to external service


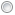
 Other


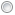
 Not applicable

If other, please specify.

- **5.** Which tool(s) are used for the WGS data analysis? (tick more than one if applicable)


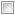
 commercial software (e.g. bionumerics)
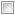
 open sourced software


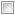
 customized pipelines composed of different tools and software


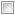
 Other


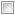
 Not applicable

If other, please specify.

## Mapping of Member States’ training needs

- **6.** In your public health reference laboratories, what are the training needs for public health operations? (tick more than one if applicable)


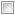
 Bioinformatic analysis


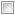
 Genome-based typing nomenclature for international comparison


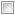
 Integration/interpretation of WGS data into public health risk assessment methods


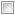
 Other

If other**,** please specify.

# Pathogens prioritised on the ECDC Roadmap - version 2.1, 2016-19

ECDC roadmap V2.1 categorises pathogens/diseases by priority for EU surveillance integration:

1. **Operationalisation of EU wide WGS-based surveillance systems in the near term:** *Listeria monocytogenes*, *Neisseria meningitidis*, Carbapenemase-producing *Enterobacteriaceae* (CPE) and antibiotic-resistant (AR) *Neisseria gonorrhoeae*.
2. **Operationalisation of WGS-based surveillance systems deferred until the required technical capacity across the EU/EEA is met:** human influenza virus, *Salmonella enterica*, Shiga-Toxin producing *E. coli* (STEC) and multidrug-resistant (MDR) *Mycobacterium tuberculosis*.
3. **Further required evidence of the opportunities and challenges:** PCR-ribotyping for *Clostridium difficile* surveillance, and sequence-based surveillance of anti-viral drug resistance in human immunodeficiency virus (HIV) and Hepatitis C virus (HCV).
4. **Postpone until next roadmap revision in 2018:** West Nile virus (WNV) and methicillin-resistant *Staphyl ococcus aureus* (MRSA).

##### For the first two above mentioned prioritization categories of pathogens (a total of 8 human pathogens), please reply to the respective tailored questions (a total of 5 questions for each pathogen).

*Listeria monocytogenes*

**7.** For the following public health operations, please define the reference laboratories WGS capacity for typing *L. monocytogenes* clinical isolates, as of July 2016:

**Control-oriented surveillance:** real-time surveillance for outbreak detection and alert

**Strategy-oriented surveillance:** trend surveillance

|  | Full capacity | Partial capacity and planning for the next 3 years (2016-18) | No current capacity but planning for the next 3 years  (2016-18) | No current capacity and no planning for the next 3 years (2016-  18) | Do not know |
| --- | --- | --- | --- | --- | --- |
| ***** Outbreak Investigation | 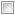 | 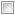 | 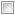 | 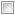 | 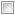 |
| ***** Control- oriented surveillance | 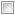 | 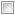 | 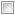 | 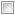 | 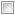 |
| ***** Strategy- oriented surveillance | 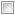 | 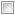 | 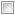 | 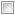 | 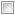 |

- **8.** Is WGS used by public health reference laboratories for typing *L. monocytogenes*, as of July 2016? (tick more than one, if applicable)


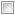
 Yes, as first line of typing


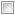
 Yes, as second line of typing, as complement


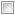
 No

Please specify the typing method(s) to which WGS complements

- **9.** Describe the sampling frame used for *L. monocytogenes* WGS typing for surveillance operations, as of July 2016:


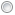
 Continuous comprehensive
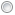
 Continuous sentinel sample


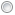
 Sampling of a subset of specimens
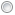
 Repeat surveys


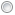
 Not applicable
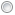
 Other

Please specify

- **10.** Describe what kind of bioinformatic analysis is(are) done for the above public health applications:

(tick more than one, if applicable)


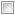
 SNP analysis
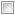
 cgMLST


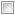
 wgMLST


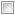
 AMR prediction


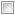
 virulence prediction
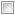
 Not applicable


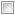
 Other

Please specify

- **11.** Where are the raw sequence data (fastq files) stored?

(tick more than one, if applicable)


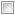
 Dedicated closed databases (national or international)

Publicly available databases (e.g. ENA), deposited along with epidemiological data (or so called "WGS metadata", e.g. country, year,...)

Publicly available databases (e.g. ENA), deposited without metadata Not applicable

If WGS data are not publicly shared, what is(are) the reason(s)?

(tick more than one, if applicable)

Personal data protection

Priority to national reporting and risk assessment

Priority to publication of national data (e.g. peer-reviewed publications) Other

Please specify the public repository database(s) used

Please specify which metadata is deposited in the public domain

*Neisseria meningitidis*

**12.** For the following public health operations, please define the reference laboratories WGS capacity for typing *N. meningitidis* clinical isolates, as of July 2016:

**Control-oriented surveillance:** surveillance for outbreak detection and alert; Identification and prevalence monitoring of vaccine escape variants, based on analysis of outer-membrane protein vaccine targets.

**Strategy-oriented surveillance:** trend surveillance (detection of emergence and spread of new virulent or epidemiologically successful sequence types) and impact assessment of immunisation programmes.

|  | Full capacity | Partial capacity and planning for the next 3 years (2016-18) | No current capacity but planning for the next 3 years  (2016-18) | No current capacity and no planning for the next 3 years (2016-  18) | Do not know |
| --- | --- | --- | --- | --- | --- |
| ***** Outbreak Investigation |  |  |  |  |  |
| ***** Control- oriented surveillance |  |  |  |  |  |
| ***** Strategy- oriented surveillance |  |  |  |  |  |

- **13.** Is WGS used by public health reference laboratories for typing *N. meningitidis*, as of July 2016?

(tick more than one, if applicable)

Yes, as first line of typing

Yes, as second line of typing, as complement No

Please specify the typing method(s) to which WGS complements

*** 14.** Describe the sampling frame used for *N. meningitidis* WGS typing for surveillance operations, as of

July 2016:

Continuous comprehensive Continuous sentinel sample

Sampling of a subset of specimens Repeat surveys

Not applicable Other

Please specify

- **15.** Describe what kind of bioinformatic analysis is(are) done for the above public health applications:

(tick more than one, if applicable)

SNP analysis

rMLST (ribosomal MLST)

MLST+*porA* VR1+*porA* VR2+*fetA*  cgMLST

AMR prediction Not applicable Other

Please specify

- **16.** Where are the raw sequence data (fastq files) stored? (tick more than one, if applicable)

Dedicated closed databases (national or international)

Publicly available databases (e.g. ENA), deposited along with epidemiological data (or so called "WGS metadata", e.g. country, year,...)

Publicly available databases (e.g. ENA), deposited without metadata Not applicable

If WGS data are not publicly shared, what is(are) the reason(s)? (tick more than one, if applicable)

Personal data protection

Priority to national reporting and risk assessment

Priority to publication of national data (e.g. peer-reviewed publications) Other

If other reason(s), please specify.

Please specify the public repository database(s) used

Please specify which metadata is deposited in the public domain

Carbapenemase-producing *Enterobacteriaceae* (CPE)

**17.** For the following public health operations, please define the reference laboratories WGS capacity for Carbapenemase-producing *Enterobacteriaceae* (CPE) typing clinical isolates, as of July 2016:

**Control-oriented surveillance:** Detection/delineation of cross-region or cross-border dissemination of high-risk clones/plasmids between repeat surveys, identification of high-prevalence geographical areas associated with spreading of specific high-risk clones and detection and genotypic identification of high-risk clones/plasmids.

**Strategy-oriented surveillance:** trend surveillance of occurrence for particular genotypes in the population and identification of high- prevalence population groups and impact assessment of prevention and control programmes.

|  | Full capacity | Partial capacity and planning for the next 3 years (2016-18) | No current capacity but planning for the next 3 years  (2016-18) | No current capacity and no planning for the next 3 years (2016-  18) | Do not know |
| --- | --- | --- | --- | --- | --- |
| ***** Control- oriented surveillance |  |  |  |  |  |
| ***** Strategy- oriented surveillance |  |  |  |  |  |

- **18.** Is WGS used by public health reference laboratories for typing CPE, as of July 2016? (tick more than one, if applicable)

Yes, as first line of typing

Yes, as second line of typing, as complement No

Please specify the typing method(s) to which WGS complements

*** 19.** Describe the sampling frame used for CPE WGS typing for surveillance operations, as of July 2016:

Continuous sentinel sample

Sampling of a subset of specimens Repeat surveys

Not applicable Other

Please specify

- **20.** Describe what kind of bioinformatic analysis is(are) done for the above public health applications:

(tick more than one, if applicable)

SNP analysis cgMLST

wgMLST

AMR prediction

Mobilome/virulome prediction Not applicable

Other

Please specify

- **21.** Where are the raw sequence data (fastq files) stored? (tick more than one, if applicable)

Dedicated closed databases (national or international)

Publicly available databases (e.g. ENA), deposited along with epidemiological data (or so called "WGS metadata", e.g. country, year,...)

Publicly available databases (e.g. ENA), deposited without metadata Not applicable

If WGS data are not publicly shared, what is(are) the reason(s)? (tick more than one, if applicable)

Personal data protection

Priority to national reporting and risk assessment

Priority to publication of national data (e.g. peer-reviewed publications) Other

If other reason(s), please specify.

Please specify the public repository database(s) used

Please specify which metadata is deposited in the public domain

Antibiotic-resistant *Neisseria gonorrhoeae*

**22.** For the following public health operations, please define the reference laboratories WGS capacity for antibiotic-resistant *N. gonorrhoeae* typing clinical isolates, as of July 2016:

**Control-oriented surveillance:** detection/delineation of emergence and cross-region/cross-border dissemination of public health relevant strains. Genotypic identification and characterisation of highly virulent, multidrug resistant and/or transmission-successful strains. Identification of high-risk patient population groups associated with the spreading of specific strains.

**Strategy-oriented surveillance:** trend surveillance of particular genotypes in the population. Understanding the dynamics of antimicrobial resistance in the context of antibiotic stewardship intervention policies.

|  | Full capacity | Partial capacity and planning for the next 3 years (2016-18) | No current capacity but planning for the next 3 years  (2016-18) | No current capacity and no planning for the next 3 years (2016-  18) | Do not know |
| --- | --- | --- | --- | --- | --- |
| ***** Control- oriented surveillance |  |  |  |  |  |
| ***** Strategy- oriented surveillance |  |  |  |  |  |

- **23.** Is WGS used by public health reference laboratories for typing antibiotic-resistant *N. gonorrhoeae*, as of July 2016? (tick more than one, if applicable)

Yes, as first line of typing

Yes, as second line of typing, as complement No

Please specify the typing method(s) to which WGS complements

*** 24.** Describe the sampling frame used for antibiotic-resistant *N. gonorrhoeae* WGS typing for surveillance operations, as of July 2016:

Continuous sentinel sample

Sampling of a subset of specimens Repeat surveys

Not applicable Other

Please specify

- **25.** Describe what kind of bioinformatic analysis is(are) done for the above public health applications:

(tick more than one, if applicable)

SNP analysis

NG-MAST (*porB* and *tbpB* genes) cgMLST

wgMLST

Mobilome/resistome/virulome prediction Not applicable

Other

Please specify

- **26.** Where are the raw sequence data (fastq files) stored? (tick more than one, if applicable)

Dedicated closed databases (national or international)

Publicly available databases (e.g. ENA), deposited along with epidemiological data (or so called "WGS metadata", e.g. country, year,...)

Publicly available databases (e.g. ENA), deposited without metadata Not applicable

If WGS data are not publicly shared, what is(are) the reason(s)? (tick more than one, if applicable)

Personal data protection

Priority to national reporting and risk assessment

Priority to publication of national data (e.g. peer-reviewed publications) Other

If other reason(s), please specify.

Please specify the public repository database(s) used

Please specify which metadata is deposited in the public domain

Influenza virus

**27.** For the following public health operations, please define the reference laboratories WGS capacity for typing Influenza virus clinical isolates, as of July 2016:

**Control-oriented surveillance:** Detection of potential pandemic influenza strains; detection of genetic change in circulating influenza viruses, vaccine strain selection and early season and season report, weekly surveillance reports (FluNewsEurope).

**Strategy-oriented surveillance:** detection of genetic markers associated with antiviral resistance and early season and season risk assessment, and vaccine effectiveness analysis.

|  | Full capacity | Partial capacity and planning for the next 3 years (2016-18) | No current capacity but planning for the next 3 years  (2016-18) | No current capacity and no planning for the next 3 years (2016-  18) | Do not know |
| --- | --- | --- | --- | --- | --- |
| ***** Outbreak Investigation |  |  |  |  |  |
| ***** Control- oriented surveillance |  |  |  |  |  |
| ***** Strategy- oriented surveillance |  |  |  |  |  |

- **28.** Is WGS used by public health reference laboratories for typing Influenza virus, as of July 2016?

(tick more than one, if applicable)

Yes, as first line of typing

Yes, as second line of typing, as complement No

Please specify the typing method(s) to which WGS complements

*** 29.** Describe the sampling frame used for influenza virus WGS typing for surveillance operations, as of

July 2016:

Continuous comprehensive

Comprehensive sentinel specimens Subset of sentinel specimens

Repeat surveys Not applicable Other

Please specify

- **30.** Describe what kind of bioinformatic analysis is(are) done for the above public health applications:

(tick more than one, if applicable)

SNP analysis

Hemagglutinin and neuraminidase sequences prediction Phylogenetic relationships

Identification of specific point mutations

Detection/prediction of antiviral resistance genetic markers Not applicable

Other

Please specify

- **31.** Where are the raw sequence data (fastq files) stored? (tick more than one, if applicable)

Dedicated closed databases (national or international)

Publicly available databases (e.g. ENA), deposited along with epidemiological data (or so called "WGS metadata", e.g. country, year,...)

Publicly available databases (e.g. ENA), deposited without metadata Not applicable

If WGS data are not publicly shared, what is(are) the reason(s)? (tick more than one, if applicable)

Personal data protection

Priority to national reporting and risk assessment

Priority to publication of national data (e.g. peer-reviewed publications) Other

If other reason(s), please specify.

Please specify the public repository database(s) used

Please specify which metadata is deposited in the public domain

#### Salmonella enterica

**32.** For the following public health operations, please define the reference laboratories WGS capacity for typing *Salmonella enterica* clinical isolates, as of July 2016:

**Control-oriented surveillance:** real-time surveillance for outbreak detection and alert

**Strategy-oriented surveillance:** trend surveillance

|  | Full capacity | Partial capacity and planning for the next 3 years (2016-18) | No current capacity but planning for the next 3 years  (2016-18) | No current capacity and no planning for the next 3 years (2016-  18) | Do not know |
| --- | --- | --- | --- | --- | --- |
| ***** Outbreak Investigation |  |  |  |  |  |
| ***** Control- oriented surveillance |  |  |  |  |  |
| ***** Strategy- oriented surveillance |  |  |  |  |  |

- **33.** Is WGS used by public health reference laboratories for typing *S. enterica*, as of July 2016?

(tick more than one, if applicable)

Yes, as first line of typing

Yes, as second line of typing, as complement No

Please specify the typing method(s) to which WGS complements

*** 34.** Describe the sampling frame used for *S. enterica* WGS typing for surveillance operations, as of July 2016:

Continuous comprehensive (all specimens, domestic and travel-associated)

Continuous comprehensive (all specimens only from domestic cases)

Continuous sentinel sample

Sampling of a subset of specimens

Repeat surveys

Not applicable

Other

Please specify

- **35.** Describe what kind of bioinformatic analysis is(are) done for the above public health applications:

(tick more than one, if applicable)

SNP analysis cgMLST

wgMLST

AMR prediction

virulence prediction mobilome prediction Not applicable

Other

Please specify

- **36.** Where are the raw sequence data (fastq files) stored? (tick more than one, if applicable)

Dedicated closed databases (national or international)

Publicly available databases (e.g. ENA), deposited along with epidemiological data (or so called "WGS metadata", e.g. country, year,...)

Publicly available databases (e.g. ENA), deposited without metadata Not applicable

If WGS data are not publicly shared, what is(are) the reason(s)? (tick more than one, if applicable)

Personal data protection

Priority to national reporting and risk assessment

Priority to publication of national data (e.g. peer-reviewed publications) Other

If other reason(s), please specify.

Please specify the public repository database(s) used

Please specify which metadata is deposited in the public domain

Shiga-Toxin producing *E. coli* (STEC)

**37.** For the following public health operations, please define the reference laboratories WGS capacity for typing STEC clinical isolates, as of July 2016:

**Control-oriented surveillance:** real-time surveillance for outbreak detection and alert

**Strategy-oriented surveillance:** trend surveillance

|  | Full capacity | Partial capacity and planning for the next 3 years (2016-18) | No current capacity but planning for the next 3 years  (2016-18) | No current capacity and no planning for the next 3 years (2016-  18) | Do not know |
| --- | --- | --- | --- | --- | --- |
| ***** Outbreak Investigation |  |  |  |  |  |
| ***** Control- oriented surveillance |  |  |  |  |  |
| ***** Strategy- oriented surveillance |  |  |  |  |  |

- **38.** Is WGS used by public health reference laboratories for typing STEC, as of July 2016?

(tick more than one, if applicable)

Yes, as first line of typing

Yes, as second line of typing, as complement No

Please specify the typing method(s) to which WGS complements

*** 39.** Describe the sampling frame used for STEC WGS typing for surveillance operations, as of July 2016:

Continuous comprehensive

Continuous sentinel sample

Sampling of a subset of specimens

Repeat surveys

Not applicable

Other

Please specify

- **40.** Describe what kind of bioinformatic analysis is(are) done for the above public health applications:

(tick more than one, if applicable)

SNP analysis cgMLST

wgMLST

AMR prediction

virulence prediction mobilome prediction Not applicable

Other

Please specify

- **41.** Where are the raw sequence data (fastq files) stored? (tick more than one, if applicable)

Dedicated closed databases (national or international)

Publicly available databases (e.g. ENA), deposited along with epidemiological data (or so called "WGS metadata", e.g. country, year,...)

Publicly available databases (e.g. ENA), deposited without metadata Not applicable

If WGS data are not publicly shared, what is(are) the reason(s)? (tick more than one, if applicable)

Personal data protection

Priority to national reporting and risk assessment

Priority to publication of national data (e.g. peer-reviewed publications) Other

If other reason(s), please specify.

Please specify the public repository database(s) used

Please specify which metadata is deposited in the public domain

Multidrug-resistant *Mycobacterium tuberculosis* (MDR-TB)

**42.** For the following public health operations, please define the reference laboratories WGS capacity for MDR-TB typing clinical isolates, as of July 2016:

**Control-oriented surveillance:** Identification and investigation of high-risk strains (‘super spreaders’ and/or MDR/XDR TB). **Strategy-oriented surveillance:** Provision of an overview of MDR TB clusters and strain diversity in the EU/EEA and Identification of high-risk geographical areas and/or population groups.

|  | Full capacity | Partial capacity and planning for the next 3 years (2016-18) | No current capacity but planning for the next 3 years  (2016-18) | No current capacity and no planning for the next 3 years (2016-  18) | Do not know |
| --- | --- | --- | --- | --- | --- |
| ***** Control- oriented surveillance |  |  |  |  |  |
| ***** Strategy- oriented surveillance |  |  |  |  |  |

- **43.** Is WGS used by public health reference laboratories for typing MDR-TB, as of July 2016? (tick more than one, if applicable)

Yes, as first line of typing

Yes, as second line of typing, as complement No

Please specify the typing method(s) to which WGS complements

*** 44.** Describe the sampling frame used for MDR-TB WGS typing for surveillance operations, as of July 2016:

Continuous comprehensive

Continuous sentinel sample

Sampling of a subset of specimens

Repeat surveys

Not applicable

Other

Please specify

- **45.** Describe what kind of bioinformatic analysis is(are) done for the above public health applications:

(tick more than one, if applicable)

SNP analysis cgMLST

drug resistance prediction Not applicable

Other

Please specify

- **46.** Where are the raw sequence data (fastq files) stored? (tick more than one, if applicable)

Dedicated closed databases (national or international)

Publicly available databases (e.g. ENA), deposited along with epidemiological data (or so called "WGS metadata", e.g. country, year,...)

Publicly available databases (e.g. ENA), deposited without metadata Not applicable

If WGS data are not publicly shared, what is(are) the reason(s)? (tick more than one, if applicable)

Personal data protection

Priority to national reporting and risk assessment

Priority to publication of national data (e.g. peer-reviewed publications) Other

If other reason(s), please specify.

Please specify the public repository database(s) used

Please specify which metadata is deposited in the public domain

# General comments to ECDC
